# Polycrystalline to preferred-(100) single crystal texture phase transformation of yttrium iron garnet nanoparticles†

**DOI:** 10.1039/c8na00123e

**Published:** 2018-09-17

**Authors:** Rameshwar B. Borade, Sagar E. Shirsath, Gaurav Vats, Anil S. Gaikwad, S. M. Patange, S. B. Kadam, R. H. Kadam, A. B. Kadam

**Affiliations:** Department of Physics, Jawahar Art Science and Commerce College Andur Osmanabad 413601 MS India; School of Materials Science and Engineering, University of New South Wales Kensington Sydney NSW 2052 Australia shirsathsagar@hotmail.com +61 469029171; Department of Physics, Vivekanand College Aurangabad 431001 MS India; Department of Physics, Materials Science Research Laboratory Shrikrishna Mahavidyalaya, Gunjoti Osmanabad 413613 MS India ram111612@yahoo.co.in +91 9423450152; Department of Physics, L.B.S. College Partur Jalna 431501 MS India

## Abstract

Nanocrystalline Ce-substituted yttrium iron garnet (YIG) powders of different compositions, Y_3−*x*_Ce_*x*_Fe_5_O_12_ (0 ≤ *x* ≤ 2.0), were synthesized by a combination of sol–gel auto-combustion and solid-state synthesis techniques. The as-obtained powder samples were sintered at 1150 °C for 10 h. The garnet structure formation is confirmed by the X-ray diffraction pattern, which shows that the calculated lattice parameter increased for *x* = 1.0 and shows a decreasing trend for *x* ≥ 1.0 with the addition of cerium ions. The lattice parameter increased from 12.38 Å to 12.41 Å for *x* ≤ 1.0 whereas it decreased from 12.412 Å to 12.405 Å with the cerium composition for *x* > 1.0. The average particle size determined by high resolution transmission electron microscopy is in the range of 50 to 90 nm and found to increase with the substitution of cerium ions in YIG. The room temperature magnetic parameters such as saturation magnetization, coercivity and remanence magnetization are greatly affected by the substitution of cerium ions. The values of saturation magnetization decrease from 25.5 to 15 emu g^−1^ whereas coercivity increases from 1 to 28 Oe with the substitution of cerium ions. The pure YIG sample shows polycrystalline nature that changed towards a single-crystal structure leading to a preferred-(100) orientation with the Ce substitution. The change from a ring to a spotty pattern observed in SAED confirmed the crystalline phase transformation and is well supported by HRTEM and magnetic measurements. The behavior of magnetic and electrical properties is well supported by the poly- and single-crystalline nature of YIG and Ce-YIG, respectively. The crystal structure transformation in YIG brought about by Ce substitution could unveil enormous opportunities in the preparation of single-crystal materials from their polycrystalline counterparts.

## Abstract 2

Nanocrystalline Ce-substituted yttrium iron garnet (YIG) powders of different compositions, Y_3−*x*_Ce_*x*_Fe_5_O_12_ (0 ≤ *x* ≤ 2.0), were synthesized by a combination of sol–gel auto-combustion and solid-state synthesis techniques.

## Graphical abstract

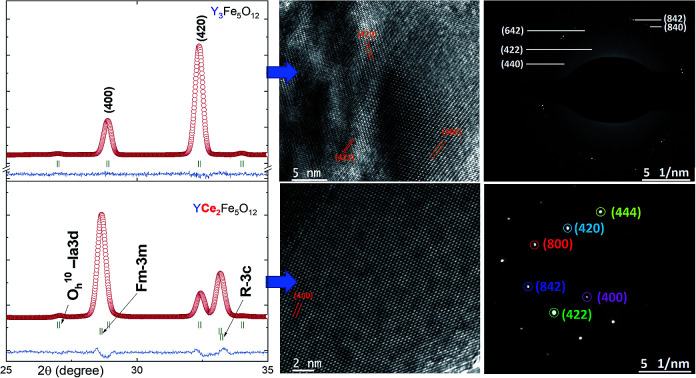

## 1. Introduction

The unique properties of single crystals are often different compared to their polycrystalline counterpart. These properties provide numerous opportunities for a variety of applications; however, manufacturing cost often precludes their widespread application. For many unique applications, such as optical lasers and X-ray scintillators, the growth of single crystals is highly complicated owing to the requirement of optimized concentrations and uniform distribution of “active” chemical dopants. It would be revolutionary and highly advantageous if single crystals of a specific compound could be cost-effectively synthesized from their polycrystalline counterpart. The crystal growth method is a common method to fabricate single crystals through solidification known as Bridgman–Czochralski processes.^1,2^ Controlled abnormal grain growth at one or several sites in the polycrystalline sample at high temperatures is also a promising methodology to grow large-sized single crystals.^3–5^ Further, the polycrystalline to single crystal growth can be achieved without passing the material through a melting stage denoted as solid-state crystal conversion.^6^ Though these methods are highly sophisticated and produce good quality single crystal materials, they are time consuming. The present work presents early attempts, results and observations on converting polycrystalline yttrium iron garnet (YIG) into preferred (100)-oriented single crystals with the combination of sol–gel autocombustion and solid-state reaction methods.

Polycrystalline YIG is a well-known ferromagnetic material that shows magneto-optic properties and is applicable in different devices, ranging from optical communications to microwave absorbers. Pure and substituted YIG are mainly applicable in microwave devices including circulators, isolators, and phase shifters because of their microwave properties such as relatively low magnetization, low dielectric loss, and narrow line width at GHz frequencies.^7,8^ YIG crystallizes in the cubic crystal system (space group *O*_h_^10^*la*3̄*d*) with an edge length of *a* = 1.24 nm.^9^ In the crystal structure, tetrahedral 24(d) and octahedral 16(a) sites are occupied by Fe^3+^ ions (3d^5^ electronic configuration) in the ratio of 3 : 2. The dodecahedral 24(c) site is occupied by non-magnetic rare earth Y^3+^ ions whereas oxygen ions occupy 96(h) sites, and therefore, the chemical equation is [Y_3_^3+^]^c^[Fe_2_^3+^]^a^(Fe_3_^3+^)^d^O_12_. The fundamental magnetic properties of YIG originate from the orientation of magnetic moments of iron ions. The magnetic moment of two octahedrally situated Fe^3+^ ions is aligned anti-parallel to that of three tetrahedrally situated Fe^3+^ ions. On the other hand, non-magnetic Y^3+^ ions are preferably situated at the dodecahedral site. Hence, the effective magnetic moment is mainly a contribution from the Fe^3+^ ions of the octahedral (a) and tetrahedral (d) sites with the association of surrounding oxygen (O^2−^) ions. In the three sites present, various ions can be substituted, and hence, the magnetic properties of YIG can be changed on a large scale. The substitution of Al or Ga ions at tetrahedral sites decreases the saturation magnetization because of the weak ferromagnetic exchange interaction developed between the octahedral a-sites and tetrahedral d-sites. The substitution of Sc or In at octahedral sites increases the magnetization, and the anisotropy can be increased by dodecahedral substitution.^10–15^ Nevertheless, limited literature has been published on trivalent ion substitution in place of Y^3+^ ions in YIG systems.^16,17^

In the present study, we replaced non-magnetic Y^3+^ ions with the non-magnetic rare earth Ce^3+^ ions in YIG and fabricated Y_3−*x*_Ce_*x*_Fe_5_O_12_ (0 ≤ *x* ≤ 2.0) nanoparticles using a combination of sol–gel auto-combustion and solid-state reaction technique. The effect of Ce^3+^ substitution on the microstructure and morphological, stoichiometric, magnetic, electrical and dielectric properties of YIG was investigated. An attempt was made to correlate the obtained properties with the polycrystalline and single crystal structure of YIG.

## 2. Experimental procedure

### 2.1 Materials

Ce-Substituted YIG (Y_3−*x*_Ce_*x*_Fe_5_O_12_; *x* = 0.0, 0.5, 1.0, 1.5, 2.0) materials were synthesized from yttrium(iii) nitrate hexahydrate [Y(NO_3_)_3_·6H_2_O] (Sigma-Aldrich, 99.9%), cerium(iii) nitrate hexahydrate [Ce(NO_3_)_3_·6H_2_O] (Acros, 99.9%), ferric(iii) nitrate nonahydrate [Fe(NO_3_)_3_·9H_2_O] (Acros, 99.9%), and citric acid monohydrate (Acros, 99.9%). Aqueous ammonia (NH_3_) solution was used to maintain the pH of the stoichiometric mixture.

### 2.2 Sample preparation

The precursor solution of pure YIG and Ce-substituted YIG was prepared by adding stoichiometric amounts of the nitrates and citric acid to 100 mL distilled water until 0.25 mol L^−1^ concentration. Citric acid as a fuel is used to chelate the ions in the aqueous solution. For making the sol neutral, ammonia in aqueous form was added dropwise continuously to the precursor solution to maintain the pH value at about 7. This solution was mixed comprehensively using a magnetic agitator until complete dissolution and heated at 90 °C for evaporation till the gel formation. The dissolution was complete in 2 h and the gel solution formed in 3 h. Again, this was dried at around 250 °C for the ignition of the gel and conversion into ash. The annealing temperature confirmed by thermogravimetric analysis/differential thermal analysis (TG/DTA) of the middle sample (*x* = 1.0) at room temperature using a thermal analyzer, model SDT Q600, was up to 1200 °C. All samples and pellets (*x* = 0.0, 0.5, 1.0, 1.5, 2.0) were annealed at 1150 °C for 10 h which were used for further study. The temperature was ramped from room temperature to 1150 °C at a heating rate of 5 °C min^−1^. In this work high temperature post-annealing is used for solid-state synthesis.

The chemical reactions of these mixtures forming YIG and Ce-substituted YIG are as follows:

1 3Y(NO_3_)_3_·6H_2_O + 5Fe(NO_3_)_3_·9H_2_O + 8C_6_H_8_O_7_·H_2_O → 3YC_6_H_5_O_7_ + 5FeC_6_H_5_O_7_ + 24HNO_3_ + 71H_2_O

2 3YC_6_H_5_O_7_ + 5FeC_6_H_5_O_7_ + O_2_ → Y_3_Fe_5_O_12_ + 20H_2_O + 48CO_2_↑

3 (3 − *x*)Y(NO_3_)_3_·6H_2_O + *x*Ce(NO_3_)_3_·6H_2_O + 5Fe(NO_3_)_3_·9H_2_O + 8C_6_H_8_O_7_·H_2_O → Y_3−*x*_Ce_*x*_Fe_5_O_12_

### 2.3 Characterization

The phase identification and crystallographic properties of YIG and Ce-substituted YIG powders were investigated using an Ultima-IV Rigaku X-ray diffractometer (XRD) with CuKα radiation, *λ* = 1.5404. Field emission scanning electron microscopy (FE-SEM; JEOL JSM-6360, MIRA-3 LMH) was employed to investigate the microstructures of the sintered samples. Elemental compositions were estimated using (x-act with INCA and AZtec EDS analysis software) X-ray electron diffraction analysis (EDAX). The particle size of the prepared nanoparticles was estimated by transmission electron microscopy (TEM; Philips CM-200). The magnetic measurements of the prepared samples were carried out using a vibrating sample magnetometer (VSM) at room temperature with the application of 0–2 T magnetic fields. Dielectric properties with an applied frequency were measured using an LCR-Q meter. The temperature dependent DC resistivity was studied by using a two-probe technique.

## 3. Result and discussion

### 3.1 Thermal analysis

**Fig. 1:**
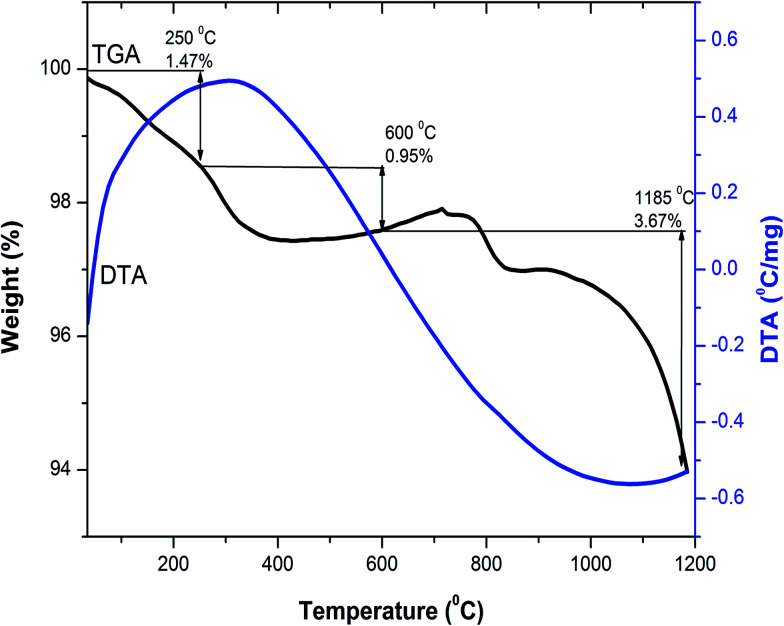
Combined differential thermal analysis (DTA) and thermogravimetric analysis (TGA) curves of Y_2_Ce_1_Fe_5_O_12_ composition.

Thermogravimetric and differential thermal analysis (TG/DTA) of the as-combusted composition Y_3−*x*_Ce_*x*_Fe_5_O_12_ (*x* = 1.0) from room temperature to 1200 °C was carried out in air atmosphere with a heating rate of 10 °C min^−1^. To investigate the probable temperature of decomposition and the phase, a combined, TG/DTA, plot of the Y_2_Ce_1_Fe_5_O_12_ composition is shown in Fig. 1. The TGA plot of the as-combusted powder synthesized by the sol–gel auto-combustion technique is classified into three steps from room temperature to 1200 °C. The first step ranging from room temperature to 250 °C corresponds to the dehydration of absorbed water. The weight loss observed in TGA in the temperature range of 250–600 °C is related to the evaporation of the residual carbon-containing compound and citric acid. The weight loss observed in the temperature range of 600–1200 °C is attributed to the decomposition of residual nitrates of [Y(NO_3_)_3_·6H_2_O], [Ce(NO_3_)_3_·6H_2_O] and [Fe(NO_3_)_3_·9H_2_O] starting materials. A total weight loss of 6% is observed in the investigated temperature range of 25–1200 °C. There are no endothermic and exothermic peaks in the temperature range from 30 °C to 1200 °C as observed from the DTA plot. The decomposition was complete at around 1100 °C, and therefore we selected 1150 °C as the sintering temperature for the Y_3−*x*_Ce_*x*_Fe_5_O_12_ (*x* = 0.0, 0.5, 1.0, 1.5, 2.0) system. The XRD analysis confirmed the synthesis of highly crystalline Ce-substituted YIG at 1150 °C.

### 3.2 Structural analysis

**Fig. 2:**
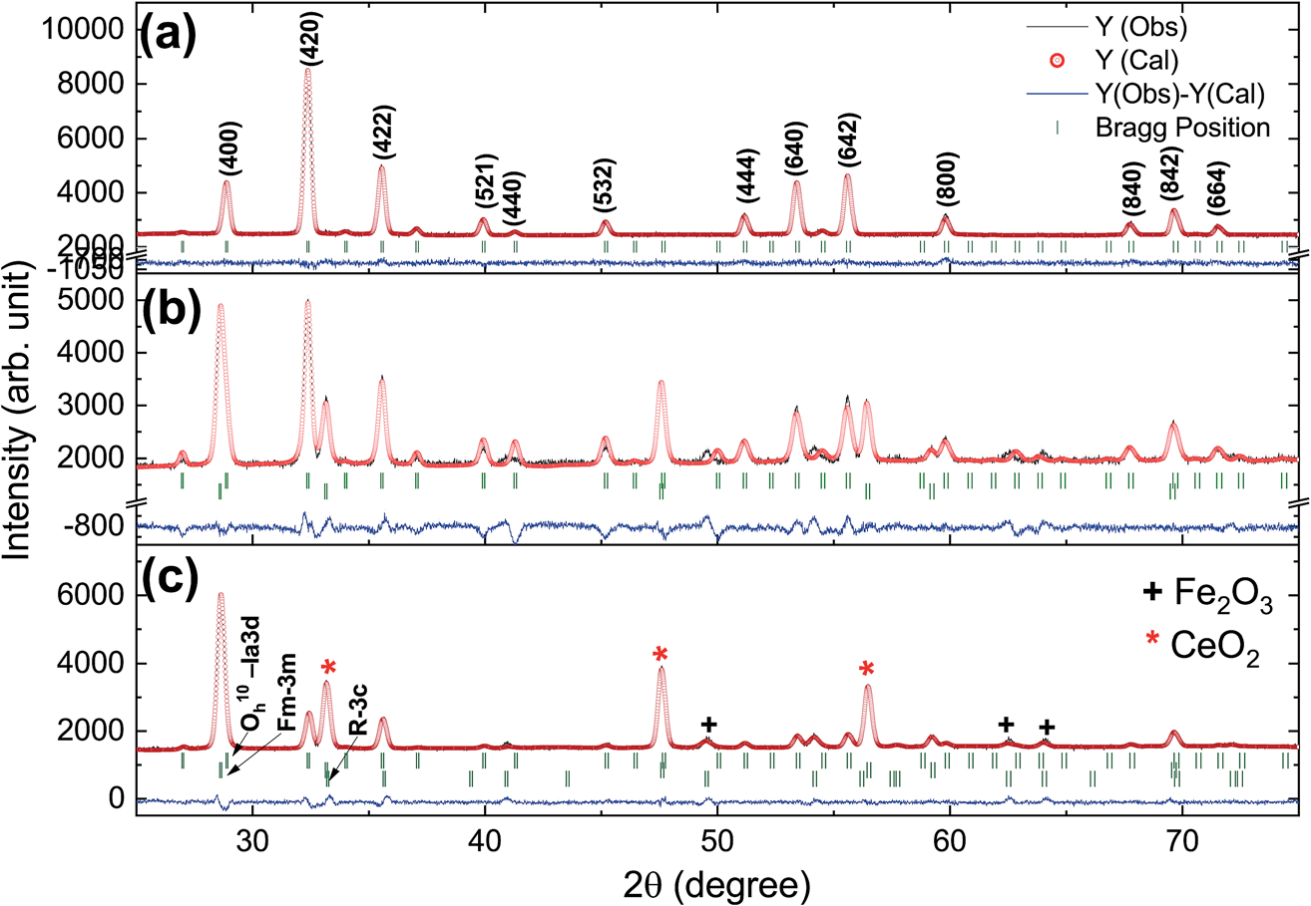
Rietveld-refined XRD patterns of typical samples of Y_3−*x*_Ce_*x*_Fe_5_O_12_ powders annealed at 1150 °C for 10 h; (a) *x* = 0.0, (b) *x* = 1.0 and (c) *x* = 2.0.

Rietveld refinement of the XRD pattern was carried out to investigate the structural changes in YIG brought about by Ce substitution. Rietveld-refined XRD patterns of the three samples are presented in Fig. 2 and the as-obtained XRD patterns of all the samples are given in the ESI (Fig. S1†). The Rietveld refinement parameters such as discrepancy factor (*R*_wp_), expected values (*R*_exp_) and goodness-of-fit factor (*χ*^2^) are presented in Table 1. The obtained peaks (400), (420), (332), (422), (521), (532), (444), (640), (642), (800), (840), (842) and (644) are in good agreement with the standard JCPDS #43-0507 data of YIG. The obtained XRD patterns exhibit single phase cubic YIG and the nanoparticles crystallize in the bcc structure. The main peak (420) of pure YIG is located at 2*θ* = 32.31° and it shifts slightly towards lower diffraction angles as Ce^3+^ content increases. Importantly, it has been observed that the intensity of the (400) peak increased whereas the intensity of the (420) peak decreased with the Ce^3+^ substitution. This variation in the peak intensity is an indication of phase changeover from polycrystalline (420) to preferred-(100) texture (400). This preferred-(100) texture phase transformation is also supported by the gradual peaking behavior of the (800) peak at 59.12° with the increase of Ce composition in YIG. The increase in (400) and (800) peak intensity is related to Ce substitution that could affect the degree of crystallization of YIG. The transformation of YIG from polycrystalline to preferred-(100) orientation may be related to the decrease in crystal plane energy or surface energy of the YIG cubic lattice with the substitution of Ce ions.^18,19^ Therefore, this transformation may be driven by a reduction in the system’s total anisotropy energy, *i.e.*, *E*_total_ = [(*γ*_s_ + *γ*_i_)/*h*] + *M*_*hkl*_*ε*^2^, where *γ*_s_, *γ*_i_ and *h* are the surface energy, interfacial energy and size of particle, respectively.^18^ The term *M*_*hkl*_*ε*^2^ is the elastic strain energy density, where *ε* and *M*_*hkl*_ are the intrinsic residual strain and the biaxial modulus, respectively, which is closely related to the crystallographic direction. However, an in-depth study needs to be carried out in the future to understand this phase transformation. The lattice parameter (*a*) was calculated by using the following equation:^20^4
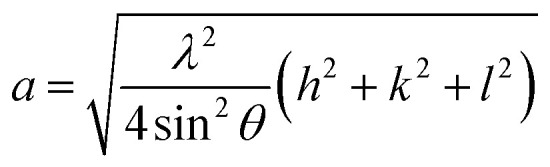


**Table 1:** Lattice constant (*a*), crystallite size (*d*_xrd_), X-ray density (*d*_*x*_), bulk density (*d*_B_), discrepancy factor (*R*_wp_), expected values (*R*_exp_) and goodness-of-fit factor (*χ*^2^) of Y_3−*x*_Ce_*x*_Fe_5_O_12_ nanoparticles. Figures in parentheses are the estimated errors

Composition (*x*)	*a* ‘Å’ (±0.002)	*d*_xrd_ ‘nm’ (±3)	*d*_x_ ‘g cm^−3^’ (±0.1)	*d*_B_ ‘g cm^−3^’ (±0.1)	*R*_exp_	*R*_wp_	*χ* ^2^
0.0	12.376	42.69	5.1598	3.077	2.79	2.13	1.29
0.5	12.391	49.51	5.3335	3.089	2.74	2.09	1.23
1.0	12.412	57.63	5.5169	3.182	3.34	2.51	1.91
1.5	12.409	65.58	5.6954	3.293	3.57	2.68	1.58
2.0	12.405	74.34	5.8749	3.405	3.36	2.54	1.34

The diffraction lines shift towards lower diffraction angles indicating that the lattice parameter increases with the substitution of the Ce^3+^ in YIG. The ionic radii of Ce^3+^ and Y^3+^ are 1.02 Å and 0.90 Å, respectively.^15^ Due to this, an enlargement of the lattice parameter would be expected with the substitution of Ce^3+^ in place of Y^3+^. The calculated values for the lattice parameter with Ce content are tabulated in Table 1. The lattice parameter increases for *x* = 1.0 but after that when *x* ≥ 1.0, the Ce^3+^ ions were hardly substituted in place of Y^3+^ ions at the dodecahedral site and hence the lattice parameter decreases. At higher levels of Ce substitution (*x* ≥ 0.5), the YIG lattice cannot accommodate Ce ions and therefore secondary phases of CeO_2_ are observed in the XRD pattern. Apart from CeO_2_ phases, the Fe_2_O_3_ phase also appeared in XRD for *x* ≥ 1.0, which is an indication of solubility limit of CeO_2_ in YIG. Also, there is lattice distortion in the structure because of the Ce^4+^ ions with higher ionic radius occupying the Y^3+^ ion sites. The values are between 12.37 Å and 12.41 Å, comparable with standard JCPDS data. The grain sizes of the YIG and Ce-substituted YIG nanoparticles were measured from the most intense peaks (400) and (420) of XRD data by the Debye–Scherrer eqn (5).^21^5
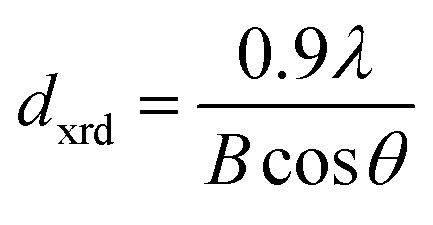
where, *d*_xrd_ is the crystallite size, *λ* is the wavelength of X-ray, *B* is the full-width at half maximum of the XRD peak taken for calculation and *θ* is the position of the XRD peak in degrees. The average crystallite size (*d*_xrd_) of the sintered samples is in the range of 42–74 nm (Table 1). The X-ray density was calculated by the following equation: 
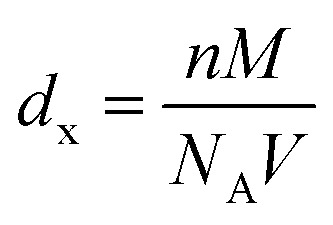
, where *M* is the molecular weight of the composition, *V* is the volume of a unit cell and *N*_A_ is the Avogadro's number. The bulk density (*d*_B_) was calculated using the Archimedes method. Calculated values of X-ray density and bulk density are listed in Table 1, which shows that the X-ray density is directly proportional to the composition of Ce in YIG. The X-ray density is also proportional to the molecular weight of the prepared samples, and the molecular weight of Y_3−*x*_Ce_*x*_Fe_5_O_12_ (*x* = 0.0, 0.5, 1.0, 1.5, 2.0) was found to increase from 737.93 g mol^−1^ to 840.35 g mol^−1^. The existence of the CeO_2_ phase also promotes the densification of YIG as Ce^4+^ ions possess high atomic mobility.^22–24^ It is assumed that the Ce-YIG forms a solid solution throughout the substitution range of Ce ions. The increase in 〈100〉 texture with the Ce substitution increases the possibility of lattice diffusion leading to an increase in the rate of cation inter-diffusion in the solid solution. The particles with a similar orientation try to diffuse together, particularly with the coinciding 〈400〉 lattice facet. Further, grain boundary diffusion is more pronounced in the grain growth as the activation energy for lattice diffusion is higher compared to that for grain boundary diffusion.^22^ The densification is further confirmed by FE-SEM.

**Fig. 3:**
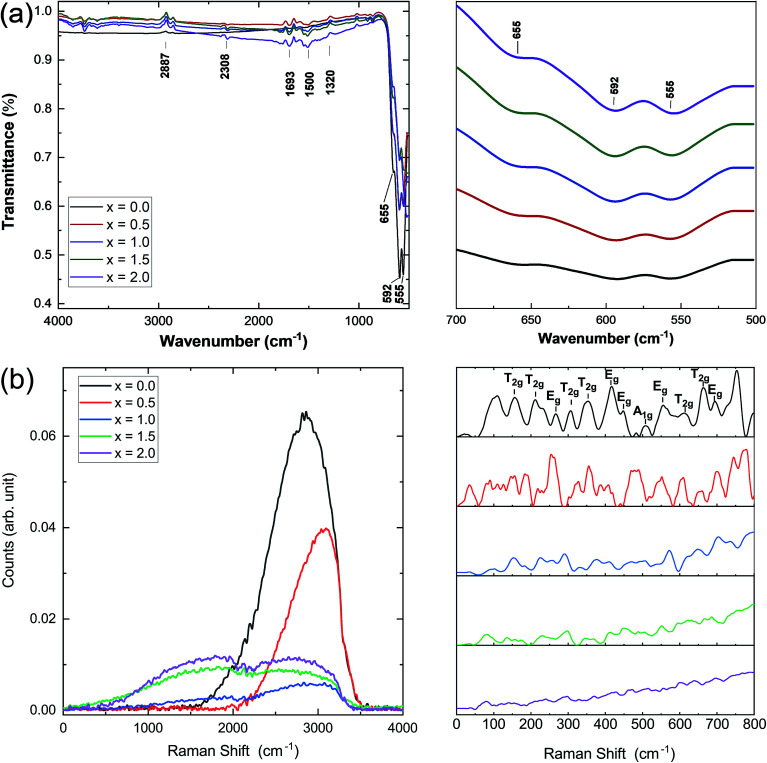
(a) Infrared spectra and (b) Raman spectra of all the Ce^3+^-substituted YIG samples.

Infrared and Raman spectroscopies were carried out on the prepared samples and the results are presented in Fig. 3. IR spectra were used to identify the band positions (Fig. 3a). It is known that the appearance of the *ν*_1_ (≈600 cm^−1^) band is attributed to the intrinsic vibrations of the octahedral group which are highly influenced by Fe–O distances.^25^ It has been observed that the bands that appeared at *ν*_1_ = 595 cm^−1^ and 558 cm^−1^ are slightly shifted to lower band frequencies at 592 cm^−1^ and 555 cm^−1^. In this study, the band frequency *ν*_1_ slightly decreases with increasing Ce concentration, whereas *ν*_2_ remains almost constant. This decrease in *ν*_1_ may be attributed to the cerium substitution of yttrium on octahedral sites. Fig. 3b shows the Raman spectra of all the samples under investigation. Well-defined peaks were observed in the Raman spectra which are in good agreement with the cubic YIG phase. (T_2g_, E_g_, and A_1g_) bands appearing in the range of 300–750 cm^−1^ are correlated with the internal modes of the free FeO_4_ tetrahedron.^26–28^ Raman bands indexed below 300 cm^−1^ were assigned to the external modes or lattice of polyhedral units; they are called as translations of tetrahedral (24d) and dodecahedral units (24c) and vibration oscillations of FeO_4_ tetrahedron. They also correspond to internal modes of the Fe^3+^ polyhedral units characterized by displacements of lighter oxygen ions.^29^ A marked difference in the Raman spectra of YIG is observed upon Ce substitution and can be related to transformation of YIG from the polycrystalline to single crystalline structure.

### 3.3 Morphological analysis

**Fig. 4:**
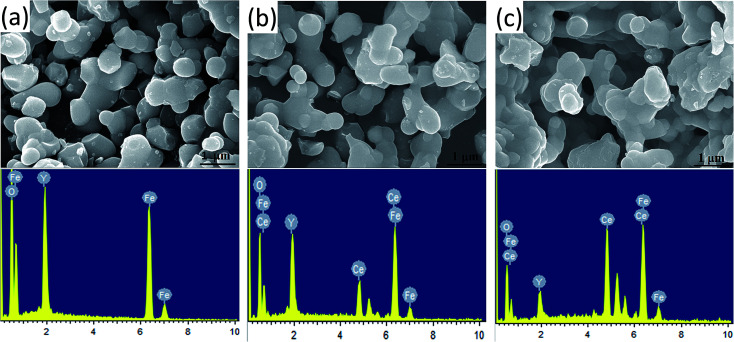
FE-SEM micrographs and the energy dispersive X-ray spectroscopy spectra of Y_3−*x*_Ce_*x*_Fe_5_O_12_, where (a) *x* = 0.0, (b) *x* = 1.0 and (c) *x* = 2.0.

The morphology of the prepared Y_3−*x*_Ce_*x*_Fe_5_O_12_ (*x* = 0.0, 1.0, 2.0) nano-crystalline powder was observed by FE-SEM, and the images of the sintered samples are given in Fig. 4. The average grain sizes are in the range of 200–300 nm. The molecular structural disorder affects the grain size and due to this a larger grain size was observed in the FE-SEM micrograph. Micrographs of the prepared samples show that most of the grains are uniform and due to the magnetic interactions some grains are agglomerated. As the Ce^3+^ substitution increases, the average grain size decreases. The decrease in grain size could be related to the compression in garnet samples because of the deformation of Fe^2+^–O^2−^–Y^3+^ and Fe^2+^–O^2−^–Fe^3+^. Also, high interfacial surface tension due to a larger surface-to-volume ratio on the nano-scale is observed. The agglomeration is the evidence of high reactivity of the composition, resulting from the magnetic interactions between the nanoparticles. As the Ce^3+^ substitution increases the percentage of agglomeration of grains also increases *i.e.* a greater number of particles are agglomerated. It is worth mentioning here that though the grain size decreased with the Ce^3+^ substitution, these small grains tend to fuse with other grains resulting in an increase in densification.

Energy-dispersive analysis of X-ray (EDAX) was employed to confirm the stoichiometric proportions and chemical composition of the selected samples of Y_3−*x*_Ce_*x*_Fe_5_O_12_ garnet nanoparticles. The selected EDAX spectra for *x* = 0.0, 1.0, 2.0 are presented in Fig. 4. The peaks of the elements Y, Ce, Fe, and O present in the EDAX pattern are evidence that the desired amount of Ce has been substituted in the YIG garnet. The compositional percentages of various elements (Y, Ce, Fe, and O) in the samples were analyzed from the EDAX pattern. The EDAX pattern shows that there is no impurity in pure and Ce-substituted YIG. The pattern indicates that the elements present in Y_3−*x*_Ce_*x*_Fe_5_O_12_ (*x* = 0.0, 1.0, 2.0) are in good stoichiometric ratios with an error of 3–4%.

**Fig. 5:**
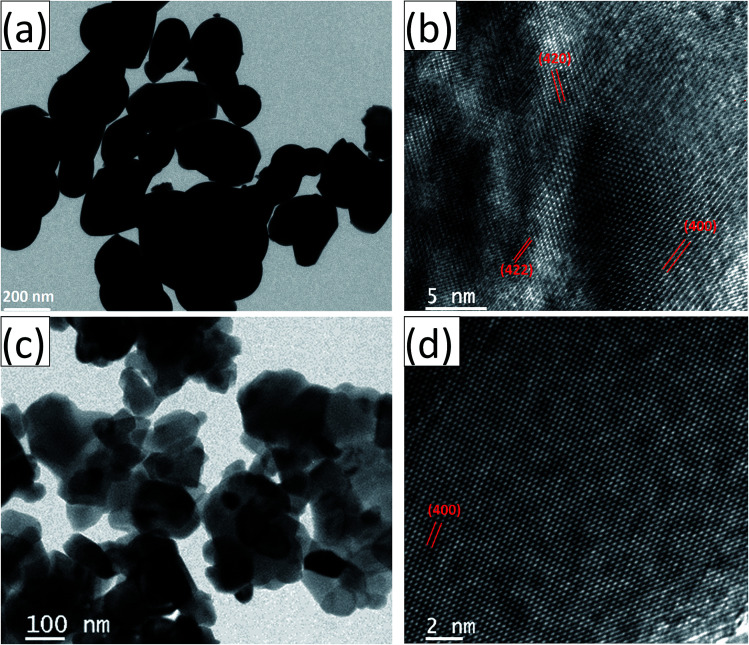
(a) and (b) are the HRTEM and high-magnification images, respectively, of polycrystalline YIG, *x* = 0.0; (c) and (d) are the HRTEM and high-magnification images, respectively, of preferred 〈100〉 oriented single-crystalline Ce-YIG, *x* = 2.0.

**Fig. 6:**
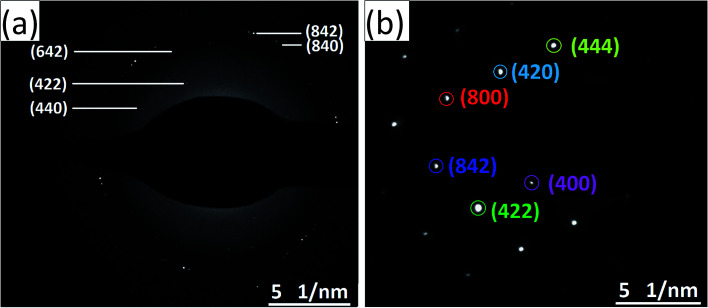
SAED patterns of (a) polycrystalline YIG, *x* = 0.0, and (b) preferred 〈100〉 oriented single-crystalline Ce-YIG, *x* = 2.0.

The HRTEM images and the corresponding high magnification images of the undoped and Ce-doped YIG are given in Fig. 5. The determination of the nanoparticles size is difficult due to overlapping of the particles. However, the average particle size of pure YIG and Ce-substituted YIG is estimated to be within the range of 50–90 nm. Fig. 5a and c indicate that the particles are not spherical in shape. This is due to the agglomeration of nanoparticles. The agglomeration occurs due to the magnetic interaction between nanoparticles during the sintering. Well-defined crystalline planes observed in the high magnification images (Fig. 5b and d) confirm the high crystallinity of the prepared sample. The crystalline planes were indexed using the *d*-spacing between them. It is obvious from Fig. 5b that there is a mixture of various planes, (400), (420) and (422), resulting from the polycrystalline nature of the sample. In contrast, Fig. 5d shows that most of the regions in the image correspond to the (400) plane confirming the preferred single-crystal structure of the sample. Fig. 6a and b show the SAED patterns of the end compositions of Y_3−*x*_Ce_*x*_Fe_5_O_12_ nanoparticles. The SAED patterns are indexed using the *d*-values and they well support the planes indexed in the XRD pattern. The obtained SAED patterns of pure and substituted YIG are remarkably different from each other. It is a known fact that polycrystalline materials show a ring pattern whereas single crystal materials show a spotty diffraction pattern in SAED. Therefore, the observed SAED patterns analogous with those of the XRD analysis confirm the transformation of pure YIG from polycrystalline nature to preferred (100)-oriented single crystals with the substitution of Ce ions.

### 3.4 Magnetic properties

**Fig. 7:**
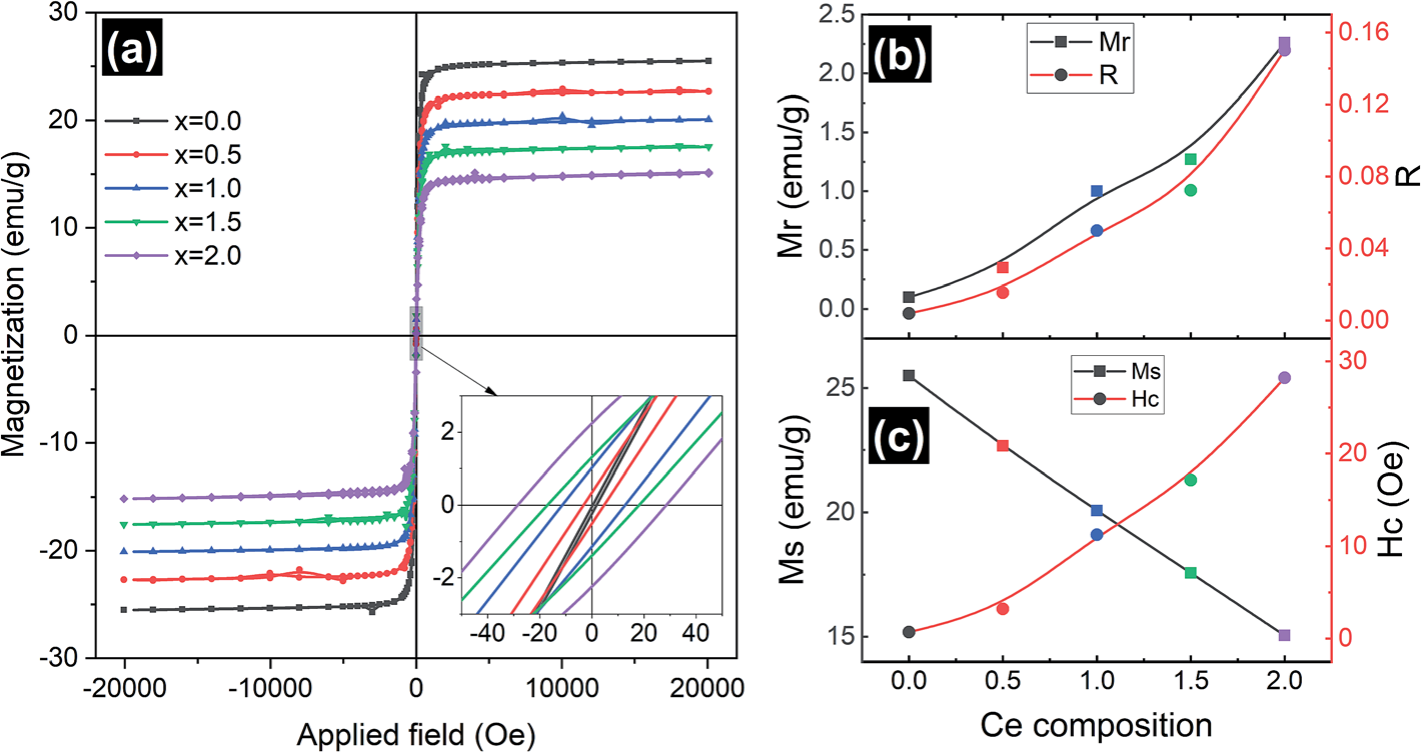
(a) Magnetic hysteresis loops; variation of magnetization with applied magnetic field, the inset shows the expanded view of MH loops at a low magnetic field; (b) variation of remanence magnetization (*M*_r_) and remanence ratio (*R*) with Ce composition and (c) variation of saturation magnetization (*M*_s_) and coercivity (*H*_c_) with Ce composition.

Magnetic measurements of the synthesized Y_3−*x*_Ce_*x*_Fe_5_O_12_ garnet samples were performed by employing a vibrating sample magnetometer by applying a magnetic field up to 2 T at room temperature and the hysteresis loops of all the compositions are presented in Fig. 7. Hysteresis graphs show narrow ‘s’ shaped loops for all the samples which indicate the soft ferromagnetic nature of the samples. Saturation magnetization (*M*_s_) decreased from 25.5 to 15 emu g^−1^ with the increase of Ce composition. This is due to the substitution of Ce^3+^ ions in place of Y^3+^ ions at the dodecahedral site. The Ce^3+^ ions are known to be paramagnetic and Y^3+^ is non-magnetic at the c-sites at room temperature, which gives rise to the canted magnetic moment of Fe^3+^ ions situated at the d-sites. Further, the appearance of the CeO_2_ phase in the XRD indicates the existence of Ce^4+^ ions which are non-magnetic in nature compared to paramagnetic Ce^3+^ ions. Further, excessive concentration of cerium results in the formation of diamagnetic Ce^4+^ ions. Electrons are absent in the 4f shell of Ce^4+^ ions and hence CeO_2_ is formed, which is non-magnetic in nature. The oxygen ions are reduced and Fe^3+^ is converted into Fe^2+^. Hence, magnetic properties decrease with increasing cerium composition. The grain size, cation substitution, and super-exchange interactions collectively determine the magnetic properties of garnet materials. In all iron garnets, Fe^3+^(a)–O^2−^–Fe^3+^(d) gives the strongest super-exchange interactions. The microscopic structure distortion of a- and d-sites is due to the large ionic radius of doped Ce^3+^ ions, which decreases super-exchange interaction and *M*_s_. The magnetic parameters such as coercivity (*H*_c_), remanence magnetization (*M*_r_) and remanence/squareness ratio (*M*_r_/*M*_s_ = *R*) extracted from the MH loops are presented in Fig. 7b and c. It is observed that remanence magnetization and coercivity of samples are very low confirming the soft magnetic nature of the Ce-YIG samples. It can be observed from Fig. 7c and the inset of Fig. 7a that *H*_c_ increases with the substitution of Ce and can be correlated with the increase in densification. The substitution of Ce in the YIG structure could also increase the magnetocrystalline, shape and magnetoelastic anisotropies causing an increase in coercivity. The values of *H*_c_ increased as cerium concentration increased because the grain size of the samples decreased. Importantly, *M*_r_ and *R* increased considerably with the increase in Ce^3+^ substitution and can be related to the preferred 〈100〉 texture orientation *i.e.* the transformation from poly- to single-crystalline nature of YIG. The increase in *R* with the transformation of YIG is an indication that 〈100〉 is the direction of magnetic easy axis of Ce-YIG.

### 3.5 Electrical properties

**Fig. 8:**
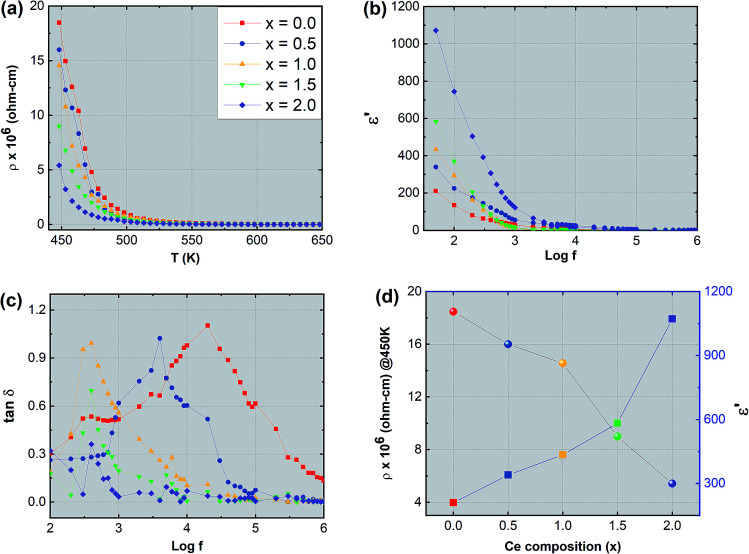
(a) Variation of electrical resistivity ‘*ρ*’ as a function of temperature ‘*T*’, (b) variation of dielectric constant ‘*ε*′’ as a function of logarithm of frequency ‘log *f*’, (c) variation in dielectric loss tangent ‘tan *δ*’ as a function of logarithm of frequency ‘log *f*’ and (d) variation of electrical resistivity ‘*ρ*’ measured at 450 K and dielectric constant ‘*ε*′’ as a function of Ce composition.

The synthesized nanoparticles of the Y_3−*x*_Ce_*x*_Fe_5_O_12_ garnet were compacted into a circular disc pellet form using a hydraulic press of dimensions ∼10 mm × 3 mm for the measurement of electrical properties by applying silver paste on both sides of the pellet for good electrical contact. Measurement of DC resistivity for all samples was carried out with a heating rate of 2 °C min^−1^ in the temperature range from 300 to 700 K. Fig. 8a reflects the variation of electrical DC resistivity as a function of temperature of all samples under investigation. Logarithm of resistivity *vs.* temperature plots for all the samples are presented in ESI Fig. S2.† The electrical DC resistivity decreases with the increase in temperature, which indicates that the Ce^3+^-substituted YIG systems possess a semiconductor-type behavior. This is in accordance with the hopping conduction mechanism where the drift mobility of electron and hole charge carriers increases with temperature. The resistivity of the garnet exhibits a Arrhenius-type temperature dependence and is given by the equation 
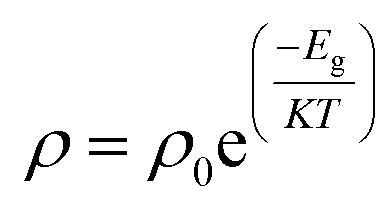
, where *k* is the Boltzmann constant and *E*_g_ is the activation energy. The variation of electrical resistivity with Ce composition is shown in Fig. 8d. DC resistivity shows a linear-type behavior with the substitution of Ce^3+^ ions in YIG, and decreased with the increase in Ce^3+^ substitution. Increase in bulk density and grain diffusion led to increased possibilities of higher mobility of electrons and *vice versa* for DC resistivity.

The complex dielectric permittivity of a dielectric substance depends on various aspects such as preparation methods, sintering temperature, microstructure and cation substitutions. Fig. 8b and c show the dielectric permittivity and dielectric loss tangent with applied frequency (50 Hz to 5 MHz) at room temperature for all samples. The dielectric permittivity decreased with applied frequency and indicates dispersion at lower frequency related to the fact that the polarization mechanism process in garnet is similar to the conducting process. Maxwell–Wagner's two-layer model explains this dispersion mechanism at low frequency^30,31^ in accordance with Koop's phenomenological theory.^32^ As the frequency increases polarization decreases and remains constant at a particular higher frequency. This can be explained as follows: at higher frequency the alternating electric field does not have any effect on electron exchange between the ferrous (Fe^2+^) ion and ferric (Fe^3+^) ion. Further, the dielectric behavior with frequency can be interpreted by using the space charge polarization which occurs because of the existence of larger conducting grains at the insulating grain boundaries.^33–38^ The conducting grains which are isolated by insulating grain boundaries give rise to space charge polarization. The variation of dielectric permittivity with Ce composition is presented in Fig. 8d. It is observed that the dielectric permittivity increases almost linearly with the increment of Ce composition. This increase in dielectric permittivity in YIG at low frequency and with Ce substitution can be a cumulative effect resulting from various factors such as ferrous (Fe^2+^) ions, oxygen vacancies, grain boundary defects *etc.* which give higher value of dielectric constant at lower frequency. In the present study the formation of the CeO_2_ phase is obvious particularly at higher substitution levels of Ce^3+^ ions in YIG. This transforms the Ce^3+^ ions into Ce^4+^ at the expense of oxygen leading to the creation of many oxygen vacancies in Ce-YIG. Such oxygen vacancies lead to two major possibilities: (i) increase in the electrical conductivity and consequently the dielectric constant and (ii) the transformation of some Fe^3+^ ions into Fe^2+^ ions to maintain the charge neutrality. It is a known fact that a material consisting of a higher percentage of Fe^2+^ ions exhibits a higher dielectric constant. The exchange of electrons among the Fe^2+^ and Fe^3+^ gives the local transportation of electrons along the external applied alternating electric field direction that gives the polarization:

6 Fe^2+^ = Fe^3+^ + e^−^

Further, in the absence of resistive grain boundaries in the Ce-YIG single crystalline type material, the high dielectric constant cannot be attributed to an internal barrier layer capacitor. Therefore, the increase in dielectric constant could be attributed to the (i) electron-pinned defect-dipoles theory, where electron hopping driven by thermal activation in defect clusters leads to high electronic conductivity, polarization and dielectric constant,^39,40^ and (ii) surface barrier layer capacitor mode that can exist in single crystals.^41,42^

Fig. 8c illustrates the variation of the dielectric loss tangent (tan δ) with applied frequency (log *f*) of Y_3−*x*_Ce_*x*_Fe_5_O_12_ at room temperature. The graph of frequency-dependent dielectric loss tangent *versus* frequency shows a maximum for all the compositions under investigation. These maxima appear due to resonance, when the frequency of the external applied alternating electric field coincides with the natural frequency of jumping ions. The maxima can only be observed if the dielectric loss tangent of the material is *ωτ* = 1 where *ω* = 2π*f*_max_ and *τ* is the relaxation time. It is observed that as Ce^3+^ content increases in YIG the maxima shift towards lower frequency and disappear at higher frequency. The maxima height decreases with increasing cerium composition in YIG. The shift of these relaxation maxima with Ce^3+^ content could be associated with the hopping of charge carriers.^43^ An increase in the number of Ce^3+^ ions increases the number of holes that may further contribute as charge carriers in Ce-YIG, and the maxima shift towards lower frequency.

**Fig. 9:**
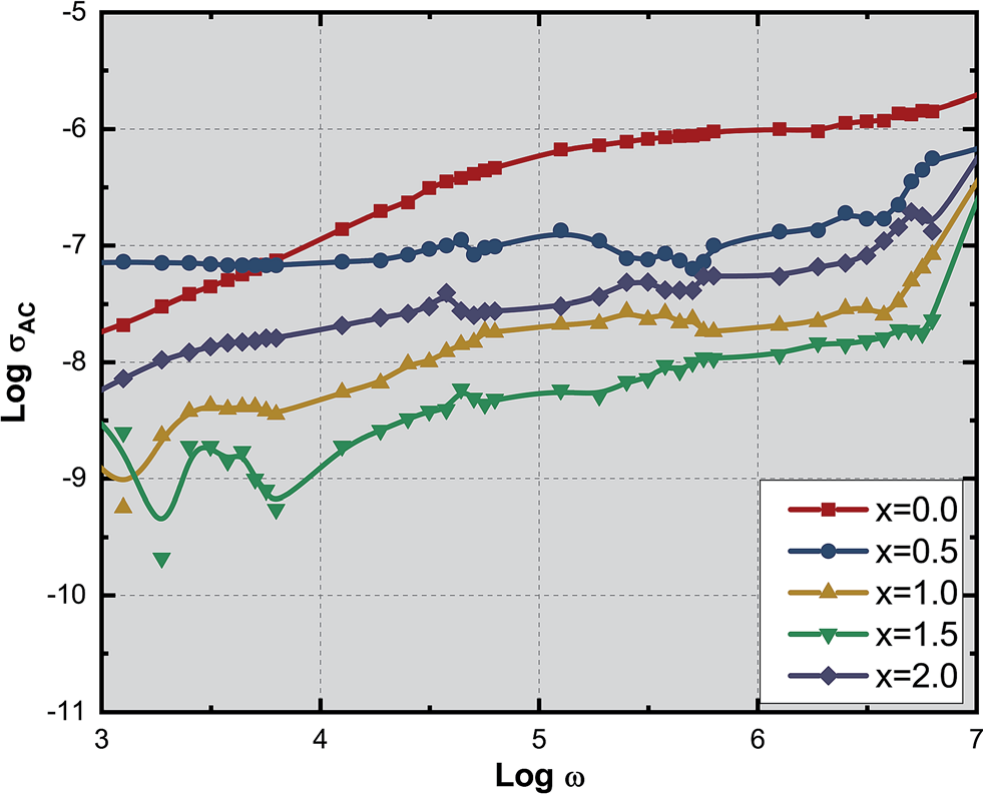
Variation of AC conductivity with frequency of all the Ce^3+^- substituted YIG samples.

Fig. 9 shows the variation of AC conductivity with frequency as a function of Ce^3+^ ion substitution in YIG at room temperature. Room temperature AC conductivity was measured with the help of dielectric data using the relation:^44^

7 *σ*_AC_ = *ωε*_0_*ε*′′

here *ε*_0_ is the permittivity of free space, *ε*′′ is the imaginary part of complex dielectric permittivity and *ω* is the angular frequency. It is found that as the frequency of the external applied field increased the AC conductivity increased linearly. This is because the increase in frequency of the external applied field improved the hopping frequency of the charge carriers. The hopping frequency depends on the angular frequency. Hence, AC conductivity increases with angular frequency. The Maxwell–Wagner two-layer model explains the AC conductivity at room temperature. The migration of electrons from Fe^2+^ and Fe^3+^ ions at octahedral sites between the adjacent layers is responsible for the conductivity in the garnet, that is the number of charge carriers available for the Fe^2+^ ions present in the garnet. These charge carriers migrate through the grains, and hence, the AC electrical conductivity increases with the frequency.

## 4. Conclusions

Ce-Substituted yttrium iron garnet nanoparticles at different concentrations have been obtained from mixed nitrate solutions by a sol–gel auto-combustion technique. The lattice parameter was found to increase with the composition of Ce until *x* = 1.0 after that it decreases with the further increase of Ce concentration. This is because of the formation of the CeO_2_ phase for the *x* > 1.0 composition. The polycrystalline random nature of YIG is slightly shifted towards the preferred-(100) texture with the increase in Ce^3+^ ions in YIG, which is observed from the change in the intensity ratio between (400) and (420) XRD peaks. The particles with a similar orientation try to diffuse together, particularly with the coinciding 〈400〉 lattice facet, leading to an increase in densification in the Ce-YIG single-crystalline material compared to its polycrystalline counterpart. The transformation from poly- to single-crystalline nature is well supported by the HR-TEM and SAED pattern, where the pure YIG shows a mixture of various planes in contrast to single-crystalline Ce-YIG where most of the regions correspond to the (400) plane confirming the preferred single-crystal structure. Pure YIG shows a ring SAED pattern whereas Ce-YIG shows a spotty diffraction pattern in SAED. Surprisingly the saturation magnetization of single-crystalline Ce-YIG is lower than that of polycrystalline YIG due to the weak ferromagnetic exchange interaction that takes place among the a–d sites and also due to the existence of nonmagnetic CeO_2_. Importantly, *M*_r_ and *R* increased considerably with the increase in Ce^3+^ substitution and can be related to the preferred 〈100〉 texture orientation *i.e.* the transformation from poly- to single-crystalline nature of YIG. The increase in *R* with the transformation of YIG is an indication that 〈100〉 is the direction of magnetic easy axis of Ce-YIG. DC resistivity decreased whereas dielectric constant increased in single-crystalline Ce-YIG and is attributed to (i) increase in densification, (ii) creation of oxygen vacancies, (iii) transformation of some Fe^3+^ ions into Fe^2+^ ions to maintain the charge neutrality, (iv) electron-pinned defect-dipoles, where electron hopping driven surface barrier layer capacitor mode can exist in Ce-YIG single crystals. The transformation of YIG from poly- to single-crystalline nature with the substitution of Ce^3+^ ions is evidenced by various characterization techniques; however, it is difficult to draw a conclusion on the major driving force behind this transformation and therefore could be a part of future studies.

## Conflicts of interest

There are no conflicts to declare.

## Supplementary Material

NA-001-C8NA00123E-s001
