# Supplementary material for: Polycrystalline to preferred-(100) single crystal texture phase transformation of yttrium iron garnet nanoparticles
Source: Nanoscale Adv. 2018 Sep 17;1(1):403–13. doi: 10.1039/c8na00123e (PMC9473261; doi:10.1039/c8na00123e)
Supplement: NA-001-C8NA00123E-s001 [file NA-001-C8NA00123E-s001.pdf]

This journal is © The Royal Society of Chemistry 2018

Supplementary Information

Polycrystalline to preferred-100 single crystal texture phase transformation of yttrium iron garnet nanoparticles

Rameshwar B. Borade [a], Sagar E. Shirsath [b,*], Gaurav Vats [b], Anil S. Gaikwad [c], S. M. Patange[d], S.B. Kadam [e], R.H. Kadam [d,*], A.B. Kadam [a]

[a] *Department of Physics, Jawahar Art Science and Commerce College, Andur, Osmanabad, 413601, MS, India*
[b] *School of Materials Science and Engineering, University of New South Wales, Kensington, Sydney, NSW 2052, Australia*
[c]*Department of Physics, Vivekanand College, Aurangabad 431001, MS, India*
[d] *Department of Physics, Materials Science Research Laboratory, Shrikrishna Mahavidyalaya, Gunjoti, Osmanabad 413613, MS, India*
[e]*Department of Physics, L.B.S. college, Partur, Jalna, 431501, MS, India*

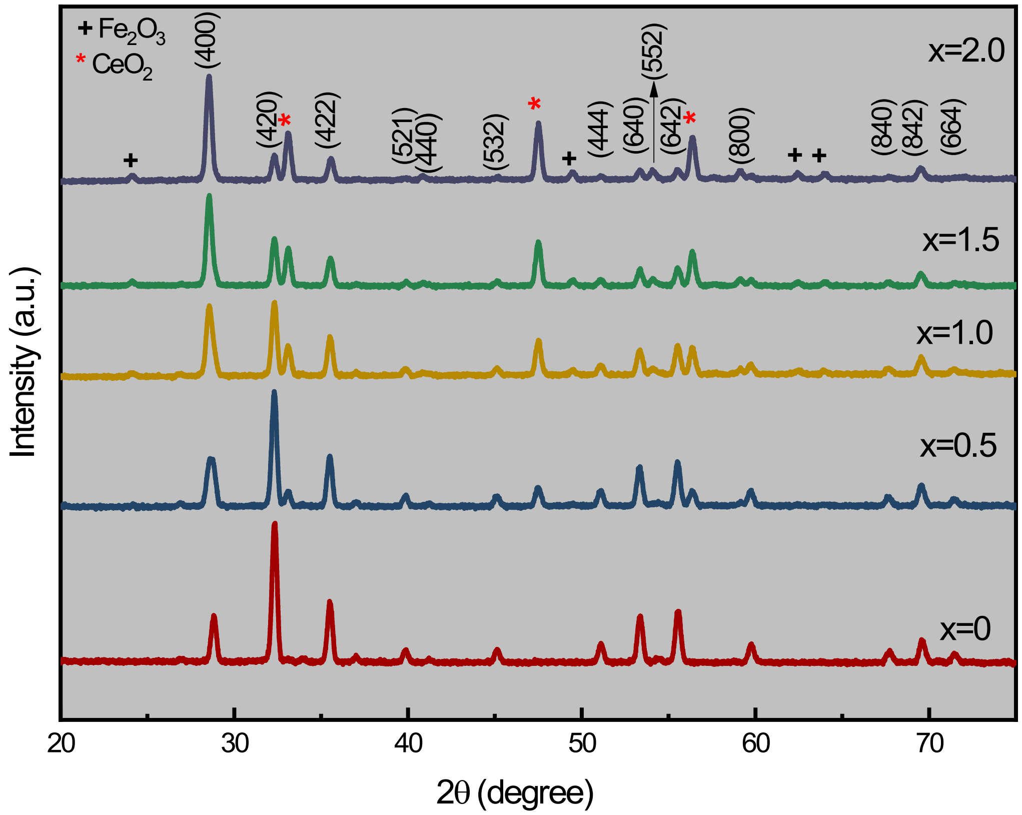

Fig. S1. XRD of $Y_{3-x}Ce_xFe_5O_{12}$ powders annealed at 1150 °C for 10 h

Activation energy:

The variation of log ρ with 1000/T are shown in Fig. S2. These plots show a sharp peak in at a particular temperature (T_{max}) at which metal insulator transition takes place. Plots exhibits metallic type behavior for T< T_{max} and insulating behavior for T> T_{max}. The Arrhenius plots divided into two regions; first is ferromagnetic and second is paramagnetic known as ordered and disordered respectively [1]. The Arrhenius plots exhibits a change in slope in each region which corresponds to the Curie point. In garnet, the charge carriers jumps from ion to ion and conduction takes place due to the electronic exchange between the ions. The activation energy in each region were calculated by Arrhenius equation in the semiconducting region (T> T_{max}). The calculated values of activation energy in the paramagnetic, ferromagnetic and their difference are listed in Table 1. The values in paramagnetic region are greater than ferrimagnetic region in

semiconducting region (T> T_{max}). The activation energy decreases with the composition of Cerium.

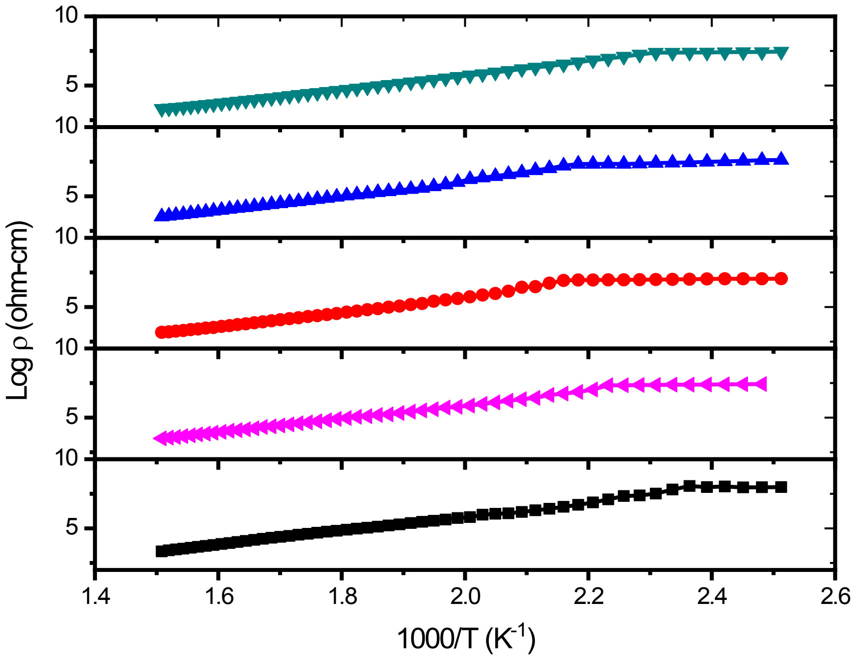

Fig. S2. Plot of logarithm of resistivity (log *ρ)* versus reciprocal of temperature (1000/T) for $Y_{3-x}Ce_xFe_5O_{12}$ samples.

Table 1

The activation energy in the paramagnetic (Ep), ferromagnetic (Ef) and their difference for $Y_{3-x}Ce_xFe_5O_{12}$ (x = 0.0, 0.5, 1.0, 1.5, 2.0) nanoparticles

Composition (x)	E_p (eV) (± 1)	E_f (eV) (± 1)	ΔE (eV) (± 0.05)
0.0	5.87	4.78	1.09
0.5	5.89	4.86	1.03
1.0	5.81	4.92	0.89
1.5	5.21	4.62	0.59
2.0	5.05	4.51	0.54

References

[1] C. Hurd, Varieties of magnetic order in solids, Contemporary Physics, 23 (1982) 469-493.
